# Aqueous supercapacitors based on carbonized silk electrodes†

**DOI:** 10.1039/c8ra01988f

**Published:** 2018-06-15

**Authors:** Limei Zhang, Zhaohui Meng, Qiaoyun Qi, Wen Yan, Naibo Lin, Xiang Yang Liu

**Affiliations:** Research Institution for Biomimetics and Soft Matter, Fujian Key Provincial Laboratory for Soft Functional Materials Research, College of Physical Science and Technology, College of Materials, Xiamen University 422 Siming Nan Road Xiamen 361005 P. R. China linnaibo@xmu.edu.cn; Department of Physics, National University of Singapore 2 Science Drive 3 Singapore 117542 Singapore phyliuxy@nus.edu.sg

## Abstract

Graphitic nitrogen-doped hierarchical porous carbon nanosheets for supercapacitor application were derived from an easily obtained and green silk by simultaneous ZnCl_2_ activation and FeCl_3_ graphitization at different heating temperatures. By increasing the heating temperature from 700 to 850 °C, the degree of graphitization and BET surface area rose to their highest levels, while the nitrogen doping content was maintained at 2.24 wt%. Carbonized silk at 850 °C displays a nanosheet morphology and a considerable specific surface area (1285.31 m^2^ g^−1^), and it was fabricated into a supercapacitor as an electrode material, exhibiting superior electrochemical performance with a high specific capacitance of 178 F g^−1^ at 0.5 A g^−1^ and an excellent rate capability (81% capacitance retention ratio even at 20 A g^−1^) in 1 mol L^−1^ H_2_SO_4_ electrolyte. A symmetric supercapacitor using carbonized silk at 850 °C as the electrodes has an excellent specific energy of 14.33 W h kg^−1^ at a power density of 251 W kg^−1^ operated over a wide voltage range of 2.0 V in aqueous neutral Na_2_SO_4_ electrolyte.

## Abstract 2

An aqueous symmetrical supercapacitor was achieved by assembling SC-850 electrodes, which possess a specific energy of 14.33 W h kg^−1^ at a power density of 251 W kg^−1^ operated over the wide voltage range of 2.0 V in aqueous neutral Na_2_SO_4_ electrolyte.

## Graphical abstract

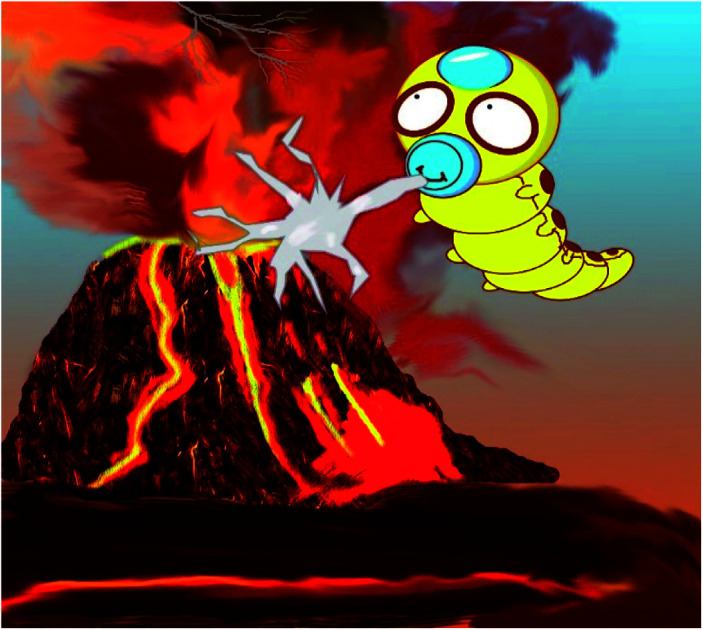

## Introduction

Supercapacitors with a high power density, a good cycle life, and low cost have attracted exceptional attention in recent years due to their extensive potential for use in the field of electrical energy storage devices.^1–6^ Supercapacitors can be divided into two types in view of their energy storage principles.^3,7–9^ One type is the electric double-layer capacitor (ECs) charge store, which originates from the typical ion adsorption at the electrode interface in the atmosphere of the electrolyte, and which demands active materials with a high accessible surface area and suitable pore size.^10^ The other type is a pseudocapacitor related to fast and highly reversible faradaic reactions using metal oxides and electrically conductive polymers as electrodes.^3,11–15^ Pseudocapacitive materials contribute to considerable capacitance, even achieving 1300 F g^−1^ (such as MnO_2_). However, their exorbitant price, lack of electrical conductivity, and disappointing cycle life have restricted their actual application.^4,9,16–19^ The drawback of supercapacitors is their very low energy density (5 W h kg^−1^ for commercial supercapacitors) relative to the battery (70–100 W h kg^−1^).^20,21^ Now the research community is trying its best to improve the energy density in various ways and from different aspects. According to the energy density formula *E* = 0.5*CU*^2^, there are undoubtedly two ways to improve the energy density of supercapacitors, as follows. On the one hand, there is improving the specific capacitance of the electrode material. There are several effective ways to improve the specific energy of a supercapacitor, including increasing the specific surface area and inducing pseudocapacitance by introducing heteroatoms.^6,10,22–25^ On the other hand, the energy density of supercapacitors can be improved by expanding the voltage window of the system by assembling an aqueous symmetric supercapacitor in a neutral electrolyte (such as Na_2_SO_4_ or Li_2_SO_4_).^26–28^ Therefore, aqueous symmetric supercapacitors based on carbon electrode materials have received considerable attention due to their lower costs and safety, and they are even easy to find relative to other types of supercapacitors. Unfortunately, they have a low energy density due to a narrow working voltage window caused by water splitting. As the Nernst equation shows, the evolution potential of the cathode is *φ*_red_ = −0.059 pH − *η*_H_2__, and the evolution potential of the anode is *φ*_ox_ = 1.23 − 0.059 pH + *η*_O_2__. If the voltage drop (IR) is taken into account, then the critical voltage window of water splitting of carbon-based symmetric supercapacitors is *E* = *φ*_ox_ − *φ*_red_ = 1.23 + *η*_H_2__ + *η*_O_2__ + IR. Therefore, to meet people's energy needs in the situation of an energy shortage, we urgently need to find a kind of carbon material that has a large oxygen evolution overpotential and a large hydrogen evolution overpotential to expand the voltage window limit and further improve the energy density.

Recently, carbon-based materials endowed with pseudocapacitive effects by doping with heteroatoms have attracted a wide range of attention. There have been many reports that nitrogen-doped porous carbon materials carbonized from natural materials with high heteroatom doping, hierarchical porosity, graphitization, and thin nanosheets can be utilized as high-storage supercapacitor electrodes. Researchers have made tremendous efforts to produce various porous carbons derived from inexpensive renewable biomass, such as sugar cane bagasse,^29^ corn husks,^30^ coconut shells,^9^ bacterial cellulose,^31^*Broussonetia papyrifera*,^32^ willow,^33,34^*Perilla frutescens*,^35^ soybean residue,^36^ flour,^37^ soybeans,^38^ human hair,^39^ and egg white.^40^ Among natural materials, silk has a high content of nitrogen (18%) and oxygen (20%);^5^ hence, it can be utilized as a carbon and nitrogen source for the production of porous carbon electrode materials.^4,6,41^ In addition, it also avoids the environmental pollution that results from achieving high nitrogen doping by the addition of a nitrogen source such as urea^34,42^ or NH_3_.^43,44^ Nitrogen-doped carbon materials from non-mulberry silk cocoons are applied in supercapacitors, which display a specific capacitance of 264 F g^−1^ in aqueous electrolytes and a stable cycle life,^46^ and which exhibit an excellent capacitance of 242 F g^−1^ in ionic liquid electrolytes.^45^ However, to the best of our knowledge, there are no reports to date regarding using the carbonization temperature to influence the porous structure, specific surface area and nitrogen content or the use of silk-derived porous carbon nanosheets to expand the voltage window of an aqueous symmetric supercapacitor in a neutral electrolyte.

In this work, silk fibroin was transformed into highly ordered graphitic carbon with considerable heteroatom doping and good electrical conductivity for use in energy conversion and storage. FeCl_3_ not only facilitates the dissolution of natural silk but also acts as a graphitization agent.^9,46^ The zinc precursor ZnCl_2_ acts as an effective activator that can introduce a porous structure with plentiful micro- and mesopores for a high surface area.^34,47^ It is expected that electrode materials prepared from low-cost and widely sourced biomass silk cocoons *via* ZnCl_2_ activation and FeCl_3_ graphitization will have excellent capacitance performance. The effects of carbonization temperature on the capacitive performance of the carbon materials will be investigated. The voltage window of a silk-derived porous carbon nanosheet based aqueous symmetric supercapacitor in a neutral electrolyte will be further expanded.

## Results and discussion

### Synthesis of carbonized silks

**Fig. 1:**
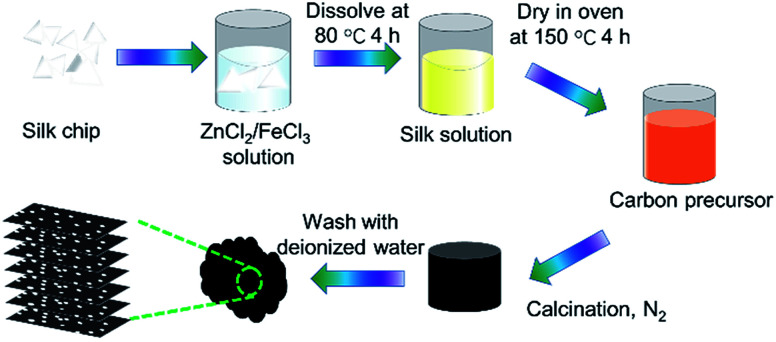
Schematic illustration for the synthesis of SCs.

Nitrogen-doped graphitic carbon-based electrode materials were prepared with ZnCl_2_ as the activator, FeCl_3_ as the graphitizer and silk as the carbon source *via* co-heat treatment. The experimental process is as follows: 3 g of *Bombyx mori* silk coccoon shells (Guangxi Sericulture Technology Co., Ltd.) was mixed with 2.5 M ferric trichloride (FeCl_3_) solution containing ZnCl_2_ (30 g). The carbon precursor was obtained by drying at 150 °C in an oven before stirring at 80 °C for 4 h. Then, the carbon precursors were put in a tubular furnace under a N_2_ atmosphere at a rate of 2 °C min^−1^ up to 700 °C, 750 °C, 850 °C, 900 °C for 1 h. Finally, acid treatment was applied to get rid of the iron species. The carbonized silks (SCs) were denoted as SC-700, SC-750, SC-850, SC-900. The preparation process is illustrated in Fig. 1.

### Morphology and structure of SCs

**Fig. 2:**
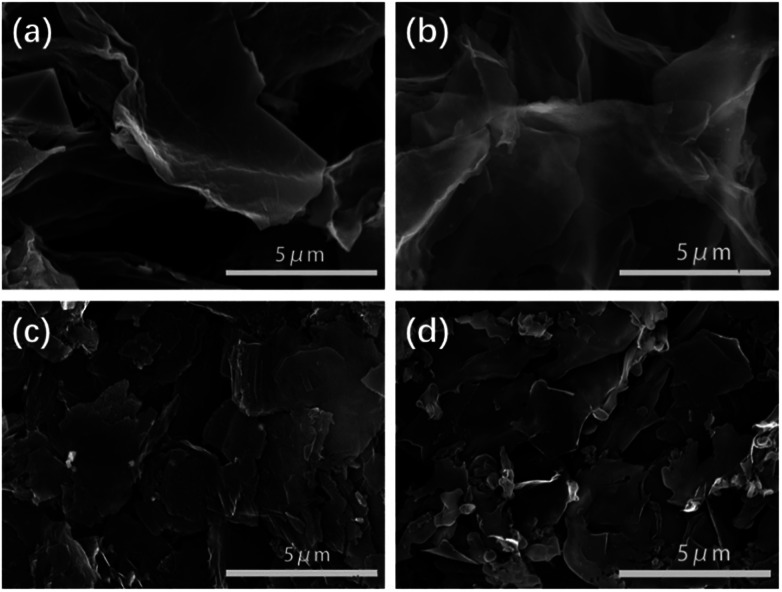
SEM images of (a) SC-700, (b) SC-750, (c) SC-850, (d) SC-900.

The pyrolysis temperature is one of the key effects on the porous structure, specific surface area and nitrogen content. SEM images of SC-700, SC-750, SC-850, SC-900 present a 2D porous nanosheet structure (Fig. 2), which is consistent with the atomic force microscopy (AFM) analysis (Fig. S1†). A porous nanosheet structure offers minimum diffusive resistance and shortens the diffusion pathways.^45^ To better observe the structure of the carbon materials, the SCs can be further viewed by high-resolution transmission electron microscopy (HR-TEM) (Fig. S2†). As shown in Fig. S2,† SC-850 and SC-900 present carbon microstructures. Nanopores can serve as active sites for ions, which contribute to the excellent capacitance and high rate performance of the electrodes.^48–53^ From HR-TEM, SC-850 has worm-like stripes, indicating the presence of partial graphitization, and SC-900 exhibits many crystal stripes, hinting at high graphitization during the carbonization process. It also shows that increasing the pyrolysis temperature favours graphitization. The conductive graphene layers can contribute fast electron transfer to the ions. With an increase in the carbonization temperature, the surfaces of the nanosheets become rougher, smaller and more fragmented. All of these factors are important in delivering excellent capacitance and high rate performance for the electrodes.

The porous structure characteristics of the SCs were further characterized by nitrogen adsorption/desorption isotherm measurements. Detailed Brunauer–Emmett–Teller (BET) specific surface areas and pore size distributions are listed in Table 1. Fig. S3† shows the pore size distribution (PSD) for SCs at different activation temperatures. SC-700, SC-750, SC-850, SC-900 exhibit a hierarchical porous structure. The micropores are mainly concentrated at about 1.2 nm; the mesopores are mainly distributed at about 3 nm. The distribution of micropores and mesopores is relatively narrow. SCs have a well-developed pore structure and a hierarchical network pore structure. This result is consistent with the pore structure information obtained by SEM and TEM. A hierarchical porous structure can provide successive channels for the transportation of ions and lessen diffusion pathways to reduce ion transport resistance.^48,52^ Abundant micro-and meso-pores provide a high surface area for ions, leading to large specific capacitance.^52,53^ Moreover, larger mesopores and nanometer-sized diffusion distances can fulfil high rate capability by rapid mass transport.^48^ The specific surface area increases with an increase in carbonization temperature. The surface area of SC-850 increases up to 1285.31 m^2^ g^−1^, probably due to the evaporation of ZnCl_2_ (boiling point = 732 °C) to create more micropores during the carbonization process. When the temperature is greater than 850 °C, the specific surface area does not change much.

**Table 1:** BET surface area, and pore size parameters of the SC samples from different temperatures (700, 750, 850 and 900 °C)

Sample	*S*_BET_ (m^2^ g^−1^)	*S*_mic_ (m^2^ g^−1^)	*S*_meso_ (m^2^ g^−1^)	*V*_total_ (cm^3^ g^−1^)	*D* (nm)
SC-900	1119.52	177.32	942.20	1.11	2.83
SC-850	1285.31	345.57	939.74	0.77	1.99
SC-750	727.76	149.44	578.32	1.10	2.51
SC-700	933.57	373.76	559.81	0.61	3.09

**Fig. 3:**
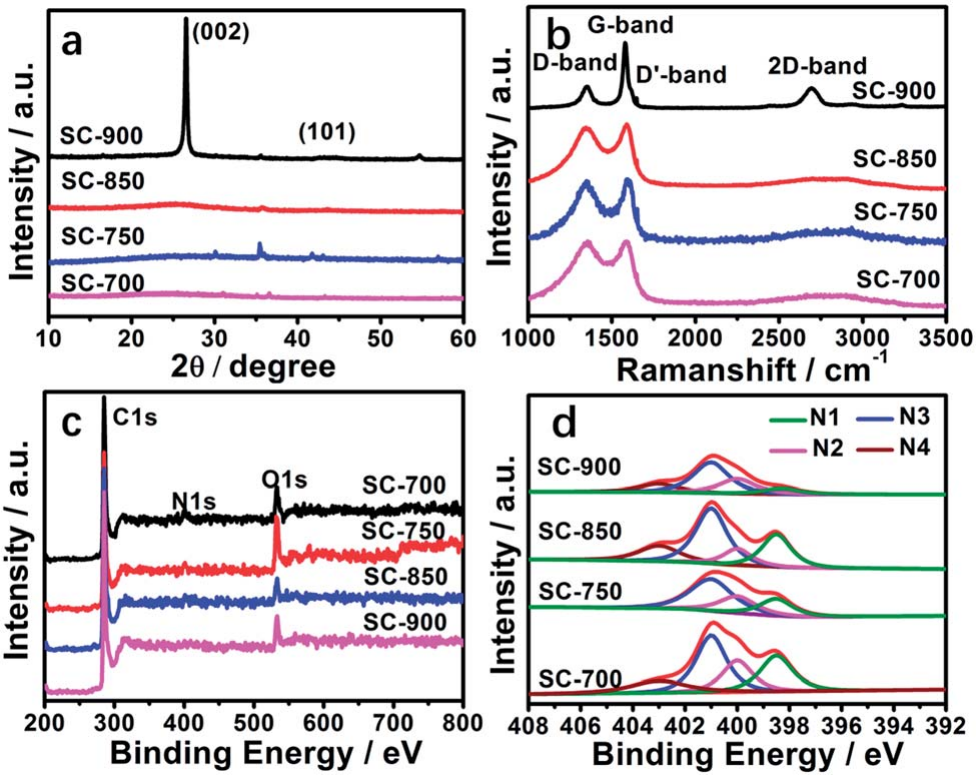
(a) XRD patterns of the SC materials, (b) Raman spectrum of SCs, (c) XPS of the SC materials, (d) N 1s XPS spectra of SC materials.

The structures of the SCs were further studied by X-ray diffraction (XRD), Raman spectroscopy and X-ray photoelectron spectroscopy (XPS) (Fig. 3, Table 2). From Fig. 3a, two characteristic diffraction peaks at 2*θ* value of 26.5° and 44° can be seen belonging to the (002), (101) planes of graphite (JCPDS card no. 41-1487).^9^ The crystallinity increases with an increase in the carbonization temperature according to the intensity of the peaks (2*θ* = 26.5°) of the SCs, indicating promotion of the degree of graphitization. This is consistent with the HR-TEM results (Fig. S2†). Meanwhile, the *d*_002_ values decreased (Table 2) due to the transformation from a stacking structure into the graphite structure.^9^ Very weak diffraction peaks at 35.5°, 54.6°, 41.6°, 43°, 56.8° belong to Fe_3_C, hinting at a few residual iron species, which is consistent with thermogravimetric (TG) analysis (Fig. S4†).

**Table 2:** XRD, Raman and XPS analysis of SCs

Sample	XRD		Raman	XPS (%)			N type (%)				
	2*θ*_002_ (deg)	*d*_002_ (nm)	*I*_G_/*I*_D_	C	N	O	N1	N2	N3	N4	N1 + N2
SC-900	26.52	0.3358	2.83	91.55	1.59	6.02	0.01	0.27	0.54	0.16	0.28
SC-850	26.48	0.3363	1.05	90.89	2.24	5.50	0.56	0.27	1.09	0.38	0.76
SC-750	26.44	0.3368	1.02	83.04	1.96	83.04	0.43	0.96	1.00	0	1.39
SC-700	26.21	0.3397	1.00	87.22	3.15	87.22	0.82	0.66	1.22	0.44	1.48

Raman spectroscopy of the SCs has three featured peaks, located in the D-band at 1351 cm^−1^ (involving defects), the D′-band at 1647 cm^−1^ (related to defects), and the G-band at 1581 cm^−1^ (related to graphitic structure) (Fig. 3b).^54–56^ The values of *I*_G_/*I*_D_ for SC-700, SC-750, SC-850, and SC-900 are 1.00, 1.02, 1.05 and 2.83, respectively (Table 2), indicating that the carbonization temperature promotes graphitization, which agrees with the XRD and HR-TEM results. Graphitization can effectively improve the electrical conductivity of carbon materials, resulting in a soaring rate capability and huge stability because it prompts charge transportation.^52,57,58^ For SC-900, the 2D band appears at 2686 cm^−1^, suggesting that a few graphene-like nanosheet structures were formed.^56^

The elemental ingredients of the SCs were further assessed by XPS. The peaks located at 284.8, 401.1, and 530.8 eV correspond to the C 1s peak of sp^2^ carbon, the N 1s peak of nitrogen, and the O 1s spectrum, respectively (Fig. 3c). The contents of C, O, N and the type of doped nitrogen obtained from XPS at different carbonization temperatures are summarized in Table 2. The high-resolution N 1s core level XPS spectra can be divided into 4 peaks (Fig. 3d) signifying pyridinic N1 (N-6 at 398.3 eV), pyridonic N2 (N-5 at 400 eV), graphitic N3 (N-Q at 401 eV) and oxidized N4 (N-X at 404 eV). As the carbonization temperature increases, the contents of N nitrogen decrease, such as pyridinic and pyridonic N type nitrogen, due to nitrogen instability and decomposition at high temperature.

### Electrochemical properties

The electrochemical performance of the SCs electrodes was characterized in 1 M H_2_SO_4_ by a three-electrode system. An ideal carbon porous structure should be able to provide a channel for rapid ion transport, which can quickly form an electric double layer at the beginning of the charge, resulting in a rectangular-like *CV* curve. The squareness of the *CV* curve reflects the diffusion rate of electrolyte ions in the carbon nanopore structure.

**Fig. 4:**
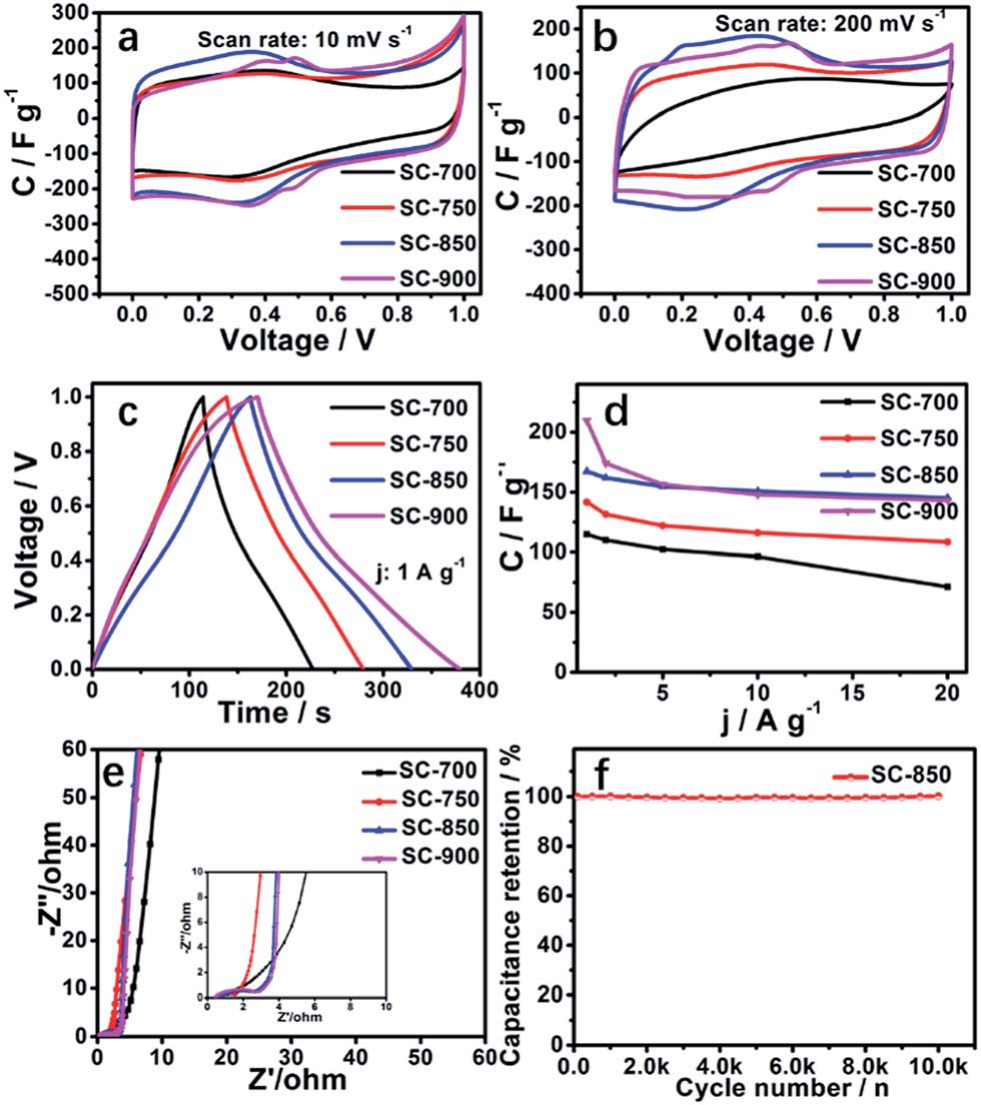
Electrochemical performance characteristics of SCs measured by a three-electrode system in 1 M H_2_SO_4_ electrolyte: (a) *CV* curves at 10 mV s^−1^; (b) *CV* curves of SCs at 200 mV s^−1^; (c) GCD curves of SCs at 1 A g^−1^; (d) specific capacitances of SCs at different current densities; (e) Nyquist plots of the SCs electrodes; (f) cycle stability curve of the SC-850 electrode.

The *CV* curves of SCs at 10 mV s^−1^ exhibit a high degree of squareness, indicating that electrolyte ions can be rapidly transported in the SC structure (Fig. 4a). Redox reaction humps located at ∼0.25 V to ∼0.5 V are related to pyridinic or pyrrolic N.^6,10,22–25,59^ While the gross contents of pyridinic and pyridonic N type nitrogen decrease with increasing carbonization temperature, the redox potential increases. At a scan rate of 200 mV s^−1^, only the *CV* curves of SC-850 and SC-900 exhibit any dramatic distortion or a large ring area (Fig. 4b). This is because SC-850 and SC-900 have large open holes and lower resistivity. The high degree of graphitization of SC-850 and SC-900 makes the materials very conductive. The *CV* curves of SC-850 and SC-900 show approximately equal encircled areas, suggesting that they have equivalent capacitance. This is because the specific surface areas of SC-850 and SC-900 are almost equal. Quasi-triangular-shaped galvanostatic charge/discharge (GCD) cycling curves further confirm the results of the *CV* curves (Fig. 4c). Fig. 4d summarizes the gravimetric specific capacitance of the SCs at current densities from 1.0 to 20 A g^−1^. Due to the slower diffusion rate of electrolyte ions in the micropores, the specific capacitance of the SCs decreases with increasing current density. As can be seen from Fig. 4d, SC-850 and SC-900 have lower specific capacitance reduction rates. The main reason is that SC-850 and SC-900 have a relatively suitable hierarchical porous network structure, which can provide channels and “buffer pools” for the rapid diffusion of ions, resulting in good performance at high magnification. Nyquist plots of SC electrodes are shown in Fig. 4e. In the low-frequency region, compared with the SC-700, SC-750 and SC-900 electrodes, SC-850 shows the straightest line with an almost 90° angle, which is characteristic of better capacitive behaviour. In the medium-frequency region, the Warburg-type line (the slope of the 45° region of the plots) of the SC-850 based supercapacitor is shorter than those of the other electrodes, which indicates that SC-850 has short electrolyte diffusion pathways due to its graphene-like structure. Although the equivalent series resistance (ESR) for SC-850 is slightly higher than that for SC-900, the obvious decrease in ion transport resistance indicates that SC-850 favours the access of ions within the electrode materials, suggesting that the porous graphene-like structure of SC-850 benefits effective ion migration into the electrode. Electrochemical impedance spectroscopy (EIS) was analysed by the software ZView 2 on the basis of the electrical equivalent circuit, as shown in Fig. S5.†*R*_s_ stands for the ionic resistance of the electrolyte, and *R*_2_ is the charge transfer resistance at the active material/current collector interface, which is caused by the faradaic reaction. *C* is related to the capacitor layer that formed during the charge–discharge process, CPE1 is related to frequency, and *W*_1_ is related to diffusion resistance. The SC-850 samples have a low ionic resistance of 0.66 Ω, and a small interfacial charge transfer resistance of 1.94 Ω. Fig. 4f is the cycle curve of SC-850; the capacitance of SC-850 shows almost no decline after 10 000 cycles at 10 A g^−1^, indicating that SC-850 has excellent electrochemical stability and reversibility.

**Fig. 5:**
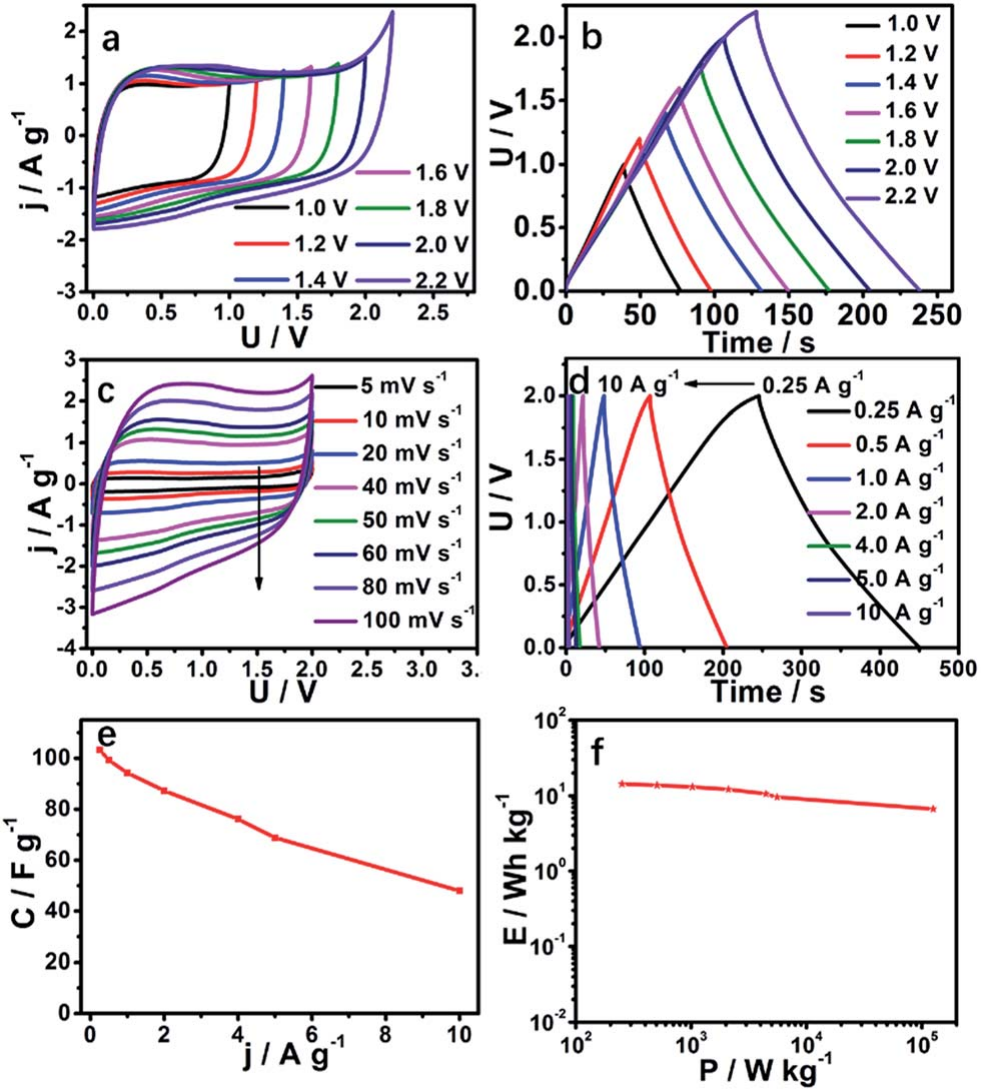
(a) *CV* curves of the symmetric SC-850 supercapacitor in different voltage windows at 50 mV s^−1^. (b) GCD curves of the symmetric SC-850 supercapacitor in different voltage windows at 0.5 A g^−1^. (c) *CV* curves of the symmetric SC-850 supercapacitor in various scan rates in a 2.0 V voltage window. (d) GCD curves of the symmetric SC-850 supercapacitor in various current densities in a 2.0 V voltage window. (e) The specific capacitance of the symmetric SC-850 supercapacitor in different current densities in a 2.0 V voltage window. (f) Ragone plots of the symmetric SC-850 supercapacitor with 1 M Na_2_SO_4_ electrolyte.

In order to study the voltage window in practical applications of SC-850, we assembled an SC-850-based symmetric supercapacitor tested by a two-electrode system in 1 M Na_2_SO_4_ aqueous electrolyte. Because we know that the energy density of a supercapacitor is proportional to the square of the voltage window, a high voltage window is expected to produce a high energy density, which is also a bottleneck in an aqueous system symmetric supercapacitor. This work has extraordinary significance for follow-up studies. The SC-850-based symmetric supercapacitor was studied at different voltage windows from 1.0 to 2.2 V (Fig. 5a). The *CV*s of the symmetric supercapacitor accompanied by a good rectangular shape possess an ideal double-layer capacitive performance, even when the voltage increases to 2.0 V. The voltage window cannot increase to 2.0 V due to the occurrence of a redox reaction. The reason why the voltage window of an SC-850-based symmetric supercapacitor can reach 2.0 V is that this carbon material has a great development of hydrogen evolution or hydrogen oxygen overpotential (Fig. S5†). The energy density of a symmetric supercapacitor was obviously improved according to the relation that the energy density is proportional to the square of the voltage window. Similarly, GCD curves (Fig. 5b) further confirm that a symmetric supercapacitor has ideal capacitive characteristics due to there being no predominant current increase even at 2.0 V. Therefore, 2.0 V was selected as the voltage window of the supercapacitor to further characterize the electrochemical performance of SC-850. The *CV*s of the SC-850 symmetric supercapacitor at different scan rates from 5 to 100 mV s^−1^ are displayed in Fig. 5c. At a high scan rate of 100 mV s^−1^, the *CV* shape is unchanged, manifesting an excellent rate capability and rapid ion transportation. Fig. 5d shows the GCD curves of the SC-850 symmetric supercapacitor at different current densities, indicating its superior capacitive behaviour and high reversibility. The SC-850-based symmetric supercapacitor possesses an electrode-specific capacitance of 103.2 F g^−1^ at a current density of 0.25 A g^−1^ (Fig. 5e). Fig. 5f shows the Ragone plots of the supercapacitor. The energy density of the SC-850 capacitor is 14.33 W h kg^−1^ at the specific power density of 251.8 W kg^−1^. The SC-850-based symmetric supercapacitor has a higher energy density relative to commercial supercapacitors (5 W h kg^−1^). The energy density of the SC-850 capacitor is much higher than the values reported for TP-NRs//ACs^60^ (13.1 W h kg^−1^), V_3_S_4_/3DGH//MnO_2_/3DGH^61^ (7.4 W h kg^−1^), NiCo_2_S_4_/graphene^62^ (2.9 W h kg^−1^) or Ni_3_S_2_–Co_9_S_8_/NF//AC^63^ (12.93 W h kg^−1^).

**Fig. 6:**
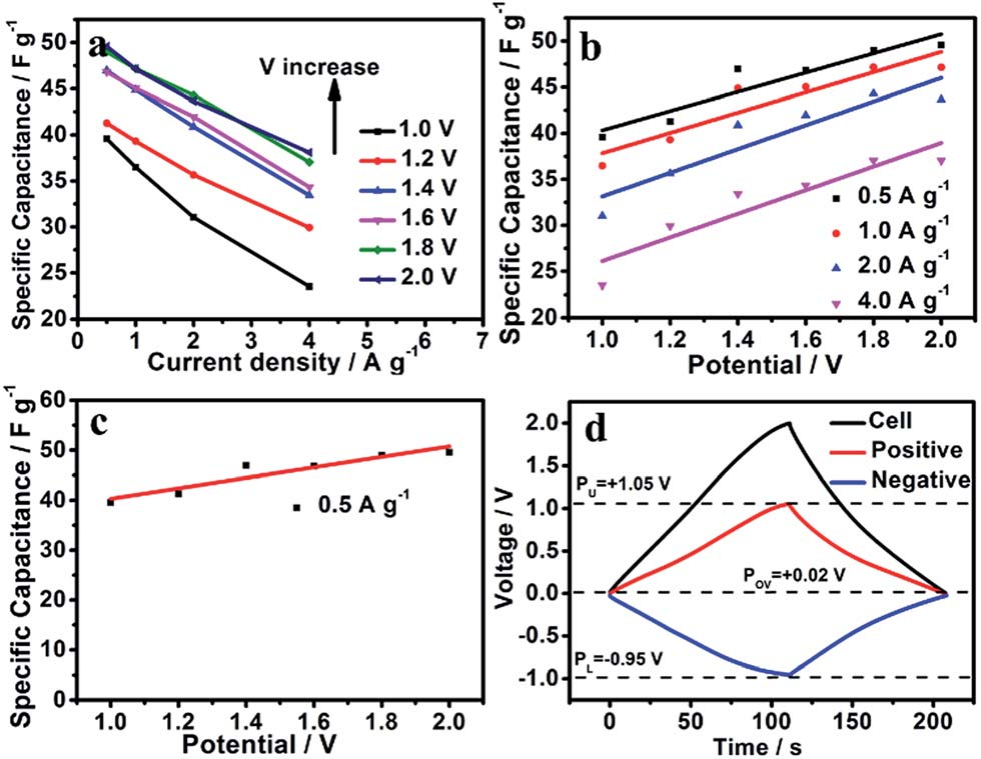
(a) Specific capacitance values for SC-850 *versus* the voltage window (V) at 0.5 A g^−1^ and linear fits. (b) *CV* curves of symmetric SC-850 with various scan rate windows at 2.0 V. (c) Specific capacitance values for SC-850 *versus* the voltage window (V) at 0.5 A g^−1^. (d) The respective potential variations of the anode and cathode when the assembled SC-850-based symmetric supercapacitors were charged/discharged at 0.5 A g^−1^ with a voltage of 2.0 V.

Nitrogen doping can improve the pseudocapacitance caused by a redox reaction. Fig. 6a shows the specific capacitance values at different current densities for different voltage windows (1.0, 1.2, 1.4, 1.6, 1.8, 2.0 V): the specific capacitance values increase with increasing voltage window. The specific capacitance (1.0 V) can provide 39.58 F g^−1^ at a low current density of 0.5 A g^−^1, whereas the specific capacitance increases to 49.58 F g^−1^ in a higher voltage window of 2.0 V. The electric double-layer capacitor makes no contribution when the voltage window is based on the storage mechanism, so the part of the capacitance that is related to the voltage window must be from pseudocapacitance.^6,23,25,59,64^ The specific capacitance for SC-850 *versus* the voltage window at different current densities shows a clear linear trend (Fig. 6b). The linear relational equation only applies in low current densities, and the double layer might affect the voltage at high current densities due to restrictive narrow pores. Herein, we select the low current density of 0.5 A g^−1^ to further analyse the experimental data. Fig. 6c displays the specific capacitance for SC-850 at 0.5 A g^−1^ over different cell voltages (1.0–2.0 V), which shows the linear relationship between capacitance and cell voltage window. The form of the relational equation of SC-850 fits:

1 *C* (F g^−1^) = *aV* + *b*

Based on the mechanism of energy storage, *b* is the double-layer capacitance (*C*_dl_) due to independence of the voltage window, while *a* is the pseudocapacitance (*C*_p_) due to dependence on the cell voltage. The relational equation of SC-850 was fitted: *C* (F g^−1^) = 10.42 V + 29.9, so *C*_dl_ = 29.9 F g^−1^ and *C*_p_ = 10.42 V. When the voltage window is 2.0 V, the double-layer capacitance is 29.9 F g^−1^, the pseudocapacitance is 20.84 F g^−1^, and the contribution of pseudocapacitance to the entire capacitance of the SC-850-based symmetric supercapacitor exceeds 70%. Doping with nitrogen enhances the performance of the materials due to the presence of pseudocapacitance, which is in line with previous research.^19,21,22^ The *CV*s of the SC-850-based symmetric supercapacitor show ideal capacitive behaviour even when the voltage extends to 2.0 V since no significant OER tail occurred (Fig. 5a). The reason why the voltage window of SC-850-based symmetric supercapacitors can reach 2.0 V is because this carbon material has a great development of hydrogen evolution or hydrogen oxygen overpotential.^28,64–66^ The conventional symmetric supercapacitors based on SC-850 electrodes were assembled with an additional reference electrode monitoring the respective potential variation of the anode and cathode when the assembled SC-850-based symmetric supercapacitors were charged/discharged at 1 A g^−1^ with a voltage of 2.0 V (Fig. 6d: the upper limit of oxygen evolution potential, PU; the lower limit of hydrogen evolution potential, PL). As shown in Fig. 6d, the practical di-hydrogen and oxygen evolution potentials at pH = 6.5 should theoretically be −0.95 V and 1.05 V, respectively. As the Nernst equation shows, the thermodynamic limit for the evolution potential of the cathode is *φ*_red_ = −0.059 pH *vs.* SHE, and the thermodynamic limit for the evolution potential of the anode is *φ*_ox_ = 1.23–0.059 pH. Thus the thermodynamic hydrogen and oxygen evolution potentials in 1 mol L^−1^ Na_2_SO_4_ (pH = 6.5) should theoretically be −0.38 V and 0.85 V, respectively. In contrast, the hydrogen evolution and oxygen evolution overpotentials widen to 0.57 V and 0.2 V, respectively. Therefore, the downward shift in *P*_0 V_ (*P*_0 V_ plays the key role in determining the potential distribution of two electrodes) would realize the possibility of further exploiting the unused energy density, then boosting the energy density of the SCs.

Considering the capacitance characteristics and synthesis costs, a supercapacitor using SC-850 as the electrodes exhibits superior performance among the four samples. This high supercapacitor performance can be attributed to the synergy effect as follows. (1) SC-850 has a hierarchical porous nanosheet nanostructure, shortening the transport length of ions and the improving rate capability. (2) The graphitized structure of SC-850 offers good electric conductivity, which also helps to reduce the electrode voltage (IR) drop. (3) SC-850 has the highest specific area (1285.31 m^2^ g^−1^). (4) SC-850 with 2.24% high N-doping not only induces pseudocapacitive behaviour, but also offers good hydrophilicity, leading to an increase in the effective access area.

## Experimental

### Characterization

X-Ray diffraction was measured by using a Bruker-AXS XRD at 2.2 kW (Cu Kα radiation, 40 kV, 20 mA). Raman spectra were carried out by using a Horiba-Jobin Yvon system with an excitation laser wavelength of 532 nm and a 100× objective lens (Numerical Aperture, NA = 0.95). The morphology of carbonized silk carbon materials was measured by an SU70 (Hitachi, Japan) scanning electron microscopy (SEM) instrument operating at 5 kV. Transmission electron microscopy (TEM, JEM-2010, Japan) was used to characterize the morphology of the carbon samples. Micromeritics Tristar II was used to measure the nitrogen adsorption–desorption isotherms at 77 K. The specific surface area of the materials was calculated by the Brunauer–Emmett–Teller (BET) theory. Surface analysis was carried out by XPS (PHI Quantum 2000 Scanning ESCA Microprobe, Physical Electronics, USA) using a monochromatic AlKα1,2.

### Electrochemical measurements

The SCs materials, acetylene black, poly (tetrafluoroethylene) (PTFE) at a weight ratio of 8 : 1 : 1 were dropped onto platinum plates and dried at 60 °C for 8 h in an oven. Ag/AgCl was used as the reference electrode, platinum foil containing graphene gel was used as the counter electrode. The capacitive performances of the SC materials were evaluated by a three-electrode system in 1 M H_2_SO_4_ aqueous electrolyte solution. The capacitive performance of the SC samples was studied by using cyclic voltammetry (*CV*, 5–100 mV s^−1^, 1.0 V), galvanostatic charge–discharge (0.25–10 A g^−1^, 1.0 V) and electrochemical impedance spectroscopy (EIS, 0.01 Hz–100 kHz, 5 mV) techniques performed on a CHI660D electrochemical workstation.

Two platinum plates containing SC-850 materials were assembled into a two-electrode system. The electrochemical workstation was used to measure cyclic voltammetry (*CV*, 5–100 mV s^−1^), galvanostatic charge–discharge (0.25–10 A g^−1^) and electrochemical impedance spectroscopy (EIS, 0.01 Hz–100 kHz, 5 mV) at different voltage windows ranging from 1.0 V to 2.4 V in 1 M Na_2_SO_4_ aqueous electrolyte solution. An additional saturated calomel electrode was added into this two-electrode system to detect the potential variation of the respective electrode *in situ*. Galvanostatic charge/discharge cycling was implemented in 1 mol L^−1^ Na_2_SO_4_ aqueous solution (pH = 6.5) using a VMP potentiostat-galvanostat (Biologic, France).

## Conflicts of interest

There are no conflicts to declare.

## Supplementary Material

RA-008-C8RA01988F-s001
