# Supplementary material for: Aqueous supercapacitors based on carbonized silk electrodes
Source: RSC Adv. 2018 Jun 15;8(39):22146–53. doi: 10.1039/c8ra01988f (PMC9081126; doi:10.1039/c8ra01988f)
Supplement: RA-008-C8RA01988F-s001 [file RA-008-C8RA01988F-s001.pdf]

Electronic Supplementary Material (ESI) for RSC Advances.
This journal is © The Royal Society of Chemistry 2018

Supplementary Information

Aqueous Supercapacitors Based on Carbonized Silk Electrodes

Limei Zhang,[a] Zhaohui Meng,[a] Wen Yan,[a] Naibo Lin,*[a] and Xiang Yang Liu*[ab]

[a] *Research Institution for Biomimetics and Soft Matter, Fujian Key Provincial Laboratory for Soft Functional Materials Research, College of Physical Science and Technology & College of Materials, Xiamen University, 422 Siming Nan Road, Xiamen 361005, P. R. China.*

[b] *Department of Physics, National University of Singapore, 2 Science Drive 3, Singapore 117542, Singapore.*

Corresponding Author

*Tel.: +86 5922182775. Fax: +86 5922182775.

To whom correspondence should be addressed:

(N. Lin) linnaibo@xmu.edu.cn

(X. Y. Liu) phyliuxy@nus.edu.sg

Notes

The authors declare no competing financial interest.

Calculations for the specific energy density (E_{cell}) and specific power density (P_{cell}) for symmetrical supercapacitors

The specific energy density (E_{cell}) and specific power density (P_{cell}) for symmetrical supercapacitors were also calculated by using eqs 1 and 2:

$$E_{cell} = \frac{C_g \Delta V^2}{8 \times 3.6} \quad (1) \qquad\qquad P_{cell} = \frac{E_{cell}}{t} \quad (2)$$

where ΔV (V) = cell voltage after ohmic drop and t (h) =discharge time.

The specific capacitance (C_g)for a single electrode was calculated by using eq 3:

$$Cg = \frac{2I}{(dV/dt)m} \quad (3)$$

where I = discharge current, dV/dt = slope measured through the discharge curve after ohmic drop, and m (g) =active material mass for a single electrode.

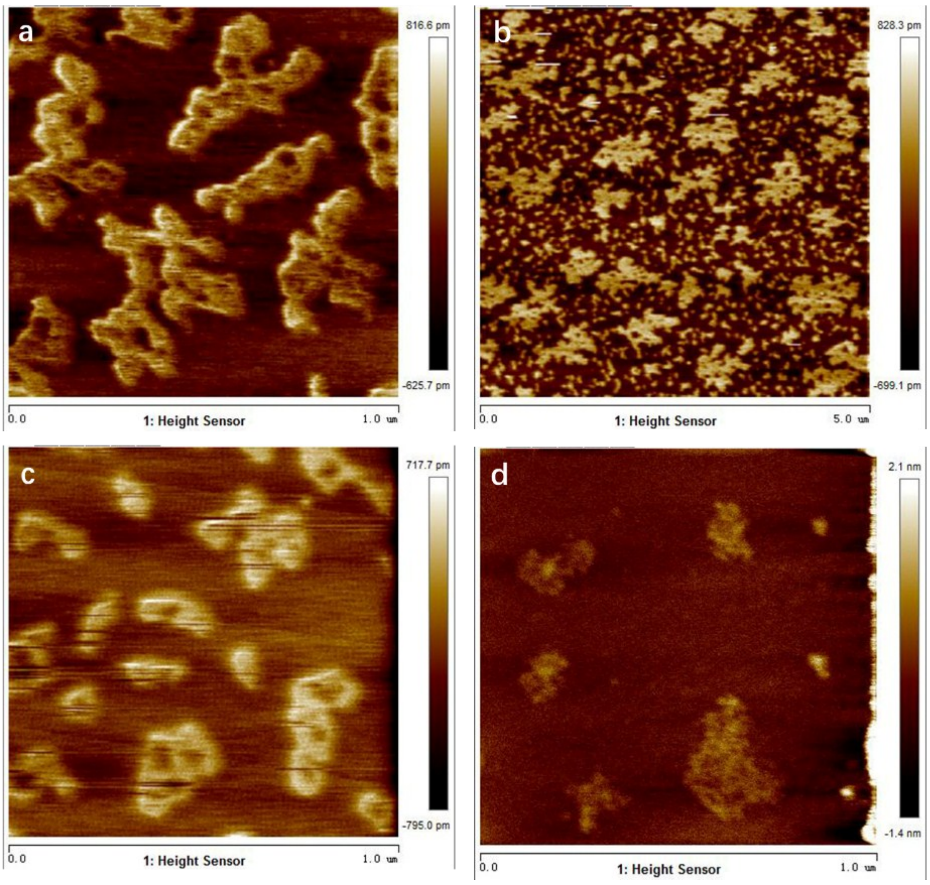

Figure S1 AFM images of (a) SC-700; (b) SC-750; (c) SC-850;(d) SC-900.

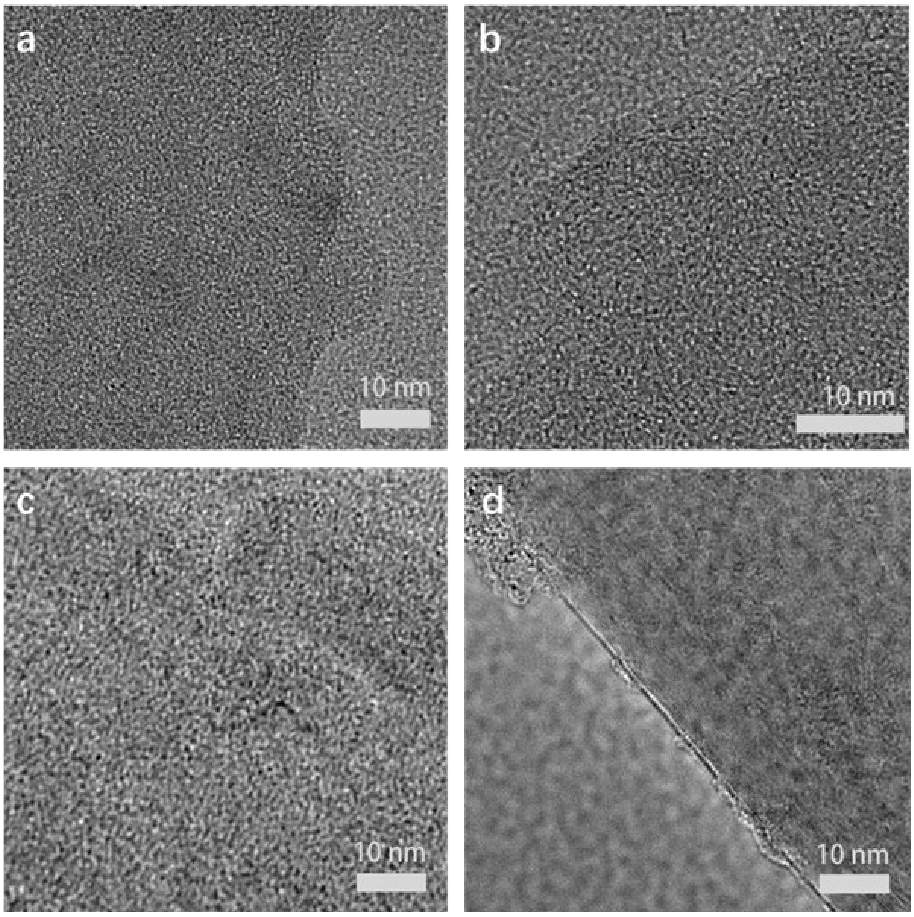

Figure S2 High-magnification TEM images of (a, b) SC-850; (c, d) SC-900.

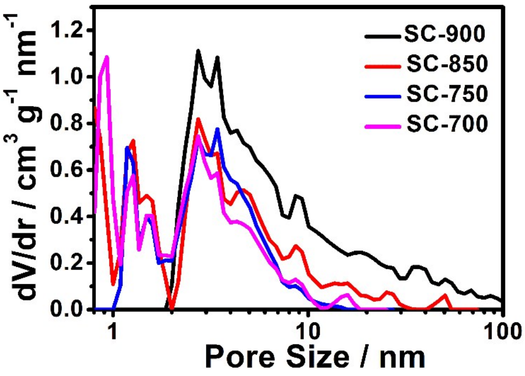

Figure S3 Pore size distribution (PSD) of SCs at different activation temperatures.

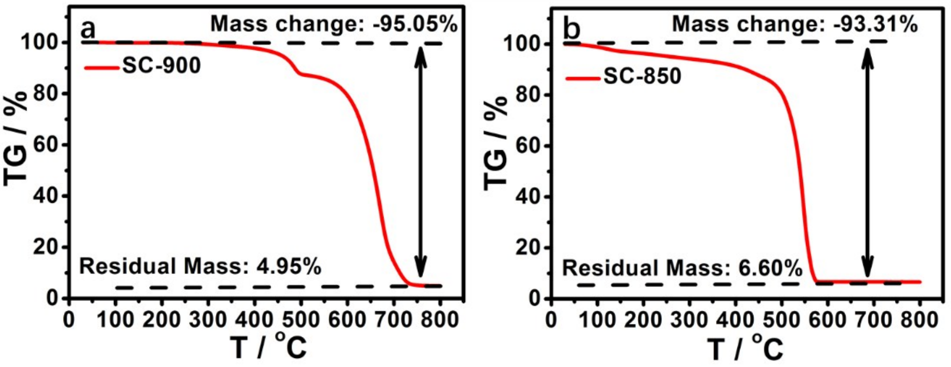

Figure S4 Thermogravimetric Analysis (TG) of SCs at different activation temperatures.

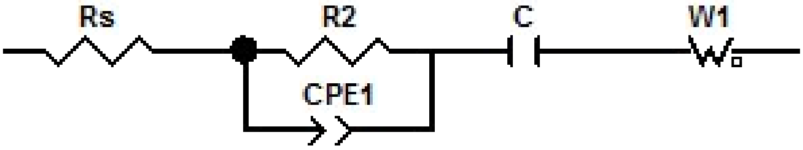

Figure S5 EIS fitting model of SCs.
